# Seipin deficiency alters brown adipose tissue thermogenesis and insulin sensitivity in a non-cell autonomous mode

**DOI:** 10.1038/srep35487

**Published:** 2016-10-17

**Authors:** L. Dollet, J. Magré, M. Joubert, C. Le May, A. Ayer, L. Arnaud, C. Pecqueur, V. Blouin, B. Cariou, X. Prieur

**Affiliations:** 1 l’institut du thorax, INSERM, CNRS, UNIV Nantes, Nantes, France; 2 Endocrinologie, CHU Caen, France; 3 CRCNA UMR_S 892, Nantes, France; 4 UMR_S 1089, Nantes, France; 5 l’institut du thorax, INSERM, CNRS, UNIV Nantes, CHU Nantes, Nantes, France

## Abstract

Loss-of-function mutations in *BSCL2* are responsible for Berardinelli-Seip congenital lipodystrophy, a rare disorder characterized by near absence of adipose tissue associated with insulin resistance. Seipin-deficient (*Bscl2*^−/−^) mice display an almost total loss of white adipose tissue (WAT) with residual brown adipose tissue (BAT). Previous cellular studies have shown that seipin deficiency alters white adipocyte differentiation. In this study, we aimed to decipher the consequences of seipin deficiency in BAT. Using a brown adipocyte cell-line, we show that seipin knockdown had very little effect on adipocyte differentiation without affecting insulin sensitivity and oxygen consumption. However, when submitted to cold acclimation or chronic β3 agonist treatment, *Bscl2*^−/−^ mice displayed altered thermogenic capacity, despite several signs of BAT remodeling. Under cold activation, *Bscl2*^−/−^ mice were able to maintain their body temperature when fed *ad libitum,* but not under short fasting. At control temperature (*i.e.* 21 °C), fasting worsened *Bscl2*^−/−^ BAT properties. Finally, *Bscl2*^−/−^ BAT displayed obvious signs of insulin resistance. Our results in these lipodystrophic mice strongly suggest that BAT activity relies on WAT as an energetic substrate provider and adipokine-producing organ. Therefore, the WAT/BAT dialogue is a key component of BAT integrity in guaranteeing its response to insulin and cold-activated adrenergic signals.

Berardinelli and Seip congenital lipodystrophy (BSCL) is a rare autosomal genetic disease characterized by an almost complete lack of white adipose tissue (WAT)^1^,^2^. BSCL is associated with metabolic disturbances, including insulin resistance, hypertriglyceridaemia, and liver steatosis. The most severe form of BSCL is caused by bi-allelic mutations in *BSCL2,* which encodes seipin, an endoplasmic reticulum (ER) protein of unknown function^3^. Seipin deficiency strongly impairs adipocyte differentiation *in vitro*^4^,^5^. In yeast and in cultured human cells, seipin deficiency alters lipid droplet (LD) morphology, with either a few giant or multiple small LDs^6^,^7^,^8^,^9^. Recently, seipin was reported to be essential for the initiation of LD formation in yeast^10^. In accordance with a potential role in triglyceride (TG) synthesis pathway, seipin was shown to interact with 1-acylglycerol-3-phosphate O-acyltransferase 2 (AGPAT2) and lipin 1^11^,^12^. Finally, seipin has been shown to promote TG storage through an interaction with the calcium pump SERCA2 in drosophila^13^. Nevertheless, the precise biological role of seipin and the exact pathways in which it is implicated remain unclear.

A major breakthrough in the understanding of the pathophysiology of BSCL2 came with the generation of global knockout (KO) mice for *Bscl2*. *Bscl2*^−/−^ mice display severe lipodystrophy, with at least a 90% decrease in WAT mass and the development of insulin resistance and hepatic steatosis, thus recapitulating the main features of the human BSCL phenotype^14^,^15^,^16^. Adipose-specific *Bscl2*^−/−^ mice exhibit progressive lipodystrophy associated with similar metabolic complications^17^, whereas the transgenic overexpression of *Bscl2* in WAT from *Bscl2*^−/−^ mice is sufficient to rescue the phenotype^18^. Finally, thiazolidinedione (TZD) treatment in global or adipose-specific *Bscl2* KO mice promotes an increase in WAT mass, leading to an improvement of the metabolic complications^16^,^17^. Together, these data demonstrate that seipin is an obligatory factor for WAT homeostasis and that seipin-deficiency in adipocytes plays a central role in the pathophysiology of BSCL.

Although the effect of seipin deficiency on WAT has been characterized, its impact on brown adipose tissue (BAT) remains unknown. BAT is the major tissue responsible for thermogenesis in mammals, burning substrates to generate heat upon adrenergic activation. In *Bscl2*^−/−^ mice, interscapular BAT is atrophied to a lower extent than WAT, with only a 60% mass reduction^14^,^15^,^16^. Seipin-deficient BAT displays an abnormal morphology with large LDs in place of typical multi-locular LDs, suggesting defective activity. However, the ability of these mice to acclimate to cold and to activate thermogenesis in BAT has not yet been explored. Interestingly, seipin-deficient gonadal WAT displays a stronger expression of the thermogenic gene encoding the uncoupling protein 1 (*Ucp1*)^19^. Accordingly, seipin deficiency is also associated with a transient induction of UCP1 expression in 3T3-L1 cells at day 4 of differentiation^19^. Collectively, these data suggest that seipin deficiency may lead to a browning of WAT and raises the question of the role of seipin in brown/beige adipocyte differentiation.

The aim of the present study was first to assess the consequence of seipin deficiency on brown adipocyte differentiation and function. We then studied the impact of seipin deficiency on BAT function *in vivo* upon cold and pharmacological stimulation. Our data demonstrated that *Bscl2*^−/−^ BAT displayed a reduced thermogenic capacity and a marked insulin resistance.

## Results

### Seipin deficiency has a minor effect on brown adipocyte differentiation and function

**Figure 1:**
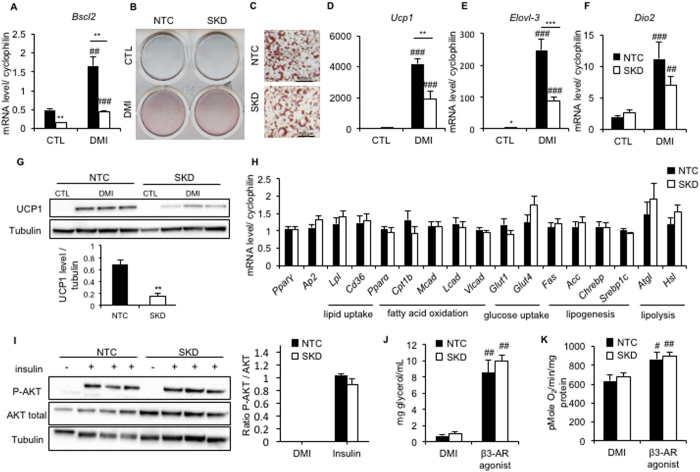
Impact of seipin deficiency in the brown adipocyte. The effects of seipin deficiency were tested using a murine brown preadipocyte cell-line, stably expressing either a non-targeted control shRNA (NTC, black bar) or a shRNA targeting *Bscl2* mRNA (SKD, white bar). (**A**) mRNA level of *Bscl2* in NTC or SKD brown adipocyte. *B-C:* Oil red O staining at macroscopic (**B**) and microscopic (**C**) levels in control (NTC) and SKD cell-lines. Scale bar 1000 μm. Expression of brown adipocyte markers *Ucp1* (**D**), *Elovl-3* (**E**) and *Dio2* (**F**) in NTC and SKD brown adipocytes cultured in the control conditions (CTL) or after eight days of differentiation (DMI). (**G**) Representative Western blot showing UCP1 expression in NTC and SKD cell-lines in the control (CTL) or differentiated (DMI) condition. Quantification of the protein levels normalized to tubulin. (**H**) Gene expression profile in eight-day differentiated NTC and SKD cells. (**I**) Western-blot quantification of phosphorylated-AKT and total AKT in differentiated NTC and SKD brown adipocytes, in absence or after insulin treatment. (**J**) Lipolysis activity estimated by measurement of glycerol release in control (black bar) and SKD (white bar) cell-lines for two hours at 37 °C with or without induction of lipolysis by the ß3AR agonist CL-316243 (100 nM). (**K**) Oxygen consumption in differentiated NTC (black bar) and SKD (white bar) brown adipocytes in the basal condition or after addition of the β3AR agonist CL-316243. All experiments were performed in triplicate, n = 3 per experiment. Bars represent SEM and significant differences between *Bscl2*^−/−^ and *Bscl2*^+/+^cells, or NTC and SKD cells, in each condition (DMI, β3AR agonist) were as follows: *p < 0.05, **p < 0.01, ***p < 0.001. Significant differences between the basal condition and the β3AR agonist-treated condition in each genotype were as follows: ^*#*^*p* < 0.05, ^*##*^*p* < 0.01, ^*###*^*p* < 0.001.

Whereas seipin deficiency has been shown to strongly impact white adipocyte differentiation in several cellular models, its effect in brown adipocytes is not yet known. To address this issue, we knockdowned *Bscl2* mRNA in an immortalized neonatal murine brown pre-adipocyte cell-line. We generated two cell-lines stably expressing a non-targeted control shRNA (NTC) or a shRNA targeting *Bscl2* mRNA (seipin knockdown: SKD). *Bscl2* mRNA expression was down-regulated by 70% in SKD *vs* NTC in both undifferentiated and differentiated states (Fig. 1A). The shRNA sequence has been previously tested in 3T3-L1 cells where it strongly impaired adipocyte differentiation with the same levels of seipin mRNA levels extinction (*data not shown*). Oil Red O staining experiments performed at day-8 of differentiation revealed similar levels of lipid-loaded adipocytes in SKD and NTC cell-lines (Fig. 1B). Moreover, no difference between SKD and NTC adipocytes morphology was visible under light microscopy (Fig. 1C). Similar results were obtained using brown pre-adipocytes isolated from 4-week old *Bscl2*^−/−^ or *Bscl2*^+/+^ mice and differentiated *in vitro*: the number of lipid loaded cells and the shape of the droplets were similar in both genotypes (Supplemental Fig. 1A). The induction of the brown adipocyte gene expression program during differentiation was then assessed in both NTC and SKD cell-lines, by comparing the non-differentiated (CTL) and 8-days differentiated (DMI) conditions (Fig. 1D–F). In both cell-lines, *Ucp1*, fatty acid elongase 3 (*Elovl-3)* and deiodinase 2 (*Dio2)* mRNA levels displayed a strong induction upon differentiation, although they were respectively reduced by 50%, 70% and 30% in SKD *vs* NTC. Similarly, UCP1 protein expression was reduced by 70% in SKD (Fig. 1G). However, mRNA expression of genes involved in adipogenesis, lipid and glucose uptake, lipid oxidation and lipogenesis was similar in SKD and NTC cells (Fig. 1H). Similar results were obtained using primary brown pre-adipocytes with a 12-fold induction of *Ucp1* mRNA levels upon differentiation in *Bscl2*^+/+^ cells compared to a 6-fold in *Bscl2*^−/−^ cells, even though this difference did not reach significance. On the other hand, *Elovl3* induction was identical in both genotypes upon differentiation (Supplemental Fig. 1B).

Then, we assessed the functional effect of seipin deficiency in brown adipocytes by testing the responses to insulin and β3 agonist stimulation. Following insulin treatment, a similar induction of Akt phosphorylation was observed in both cell-lines, indicating no alteration in insulin signaling (Fig. 1I). To further assess brown adipocyte functionality, the lipolytic response (Fig. 1J) and oxygen consumption using the Seahorse system (Fig. 1K) were measured upon β3AR agonist (CL-316243) stimulation. The induction levels were similar in SKD and NTC cell-lines, both in the basal state and in response to CL-316243 which increased glycerol release by 10 times (from 0.73 ± 0.19 to 8.55 ± 1.52 mg glycerol/mL in NTC cells; from 0.99 ± 0.25 to 10.01 ± 0.68 mg glycerol/mL in SKD cells), and oxygen consumption by 35% and 32% in NTC and SKD cells respectively (638 ± 60 to 863 ± 80 pMole O2/min/mg protein in NTC cells *vs* 681 ± 38 to 900 ± 43 pMole O2/min/mg protein in SKD cells). As for the insulin signaling, *Bscl2*^−/−^ or *Bscl2*^+/+^ primary adipocytes displayed the same responses (Supplemental Fig. 1C) Taken together, these *in vitro* results indicate that seipin deficiency modestly alters the induction of thermogenic genes during differentiation but without important functional consequences on oxygen consumption and lipolysis.

### *Bscl2* ^−/−^ mice can cope with cold acclimation but display a reduced maximal thermogenic capacity

**Figure 2:**
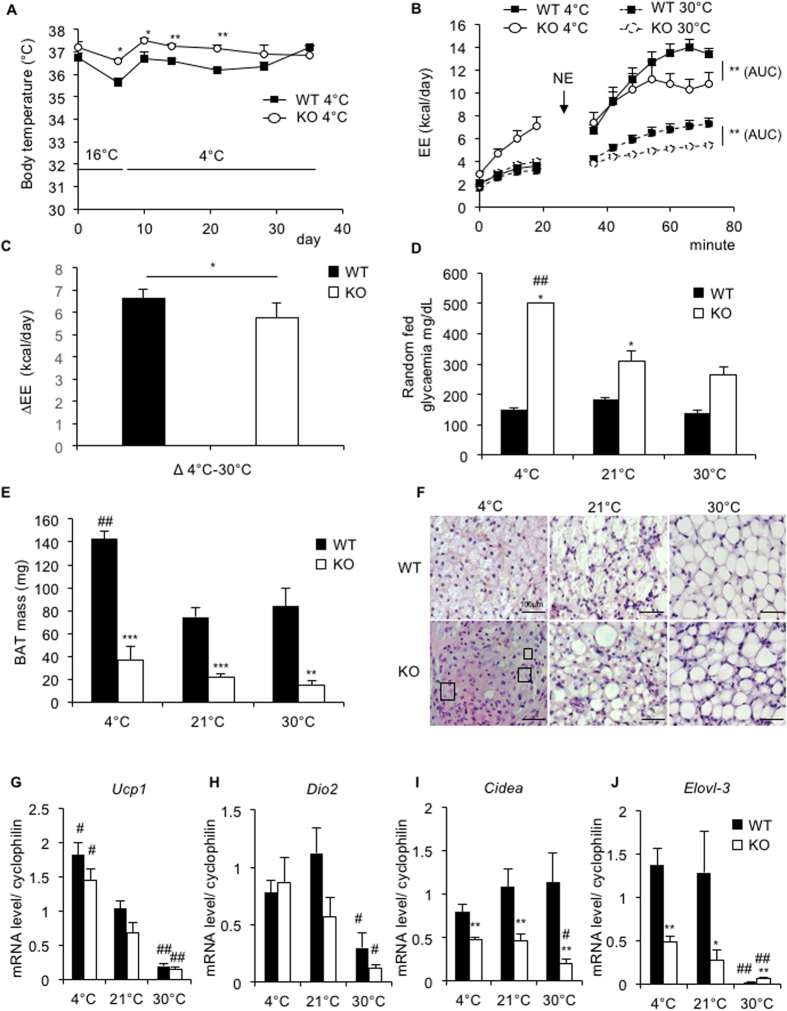
Adaptation of *Bscl2*^+/+^ and *Bscl2*^−/−^ mice to temperature challenges. Nine-week-old *Bscl2*^+/+^ and *Bscl2*^−/−^ mice were maintained in a cold environment (one week at 16 °C followed by four weeks at 4 °C), thermoneutral environment (five weeks at 30 °C) or at control temperature (21 °C). *Bscl2*^+/+^ (WT, solid line, black bar) and *Bscl2*^−/−^ (KO, dashed line, white bar) mice. (**A**) Body temperature measurement during cold acclimation. (**B**) In order to assess the maximal thermogenic capacity, energy expenditure was measured using metabolic cages in cold-acclimated *Bscl2*^+/+^ (solid line, black square) and *Bscl2*^−/−^ (solid line, white circle) mice, and in 30°-acclimated *Bscl2*^+/+^ (dashed line, black square) and *Bscl2*^−/−^ (dashed line, white circle) mice, before (t 0–20 min) and after (t 35–75 min) norepinephrine injection. (**C**) Maximum thermogenic capacity is defined by the difference in *Bscl2*^−/−^ and in to *Bscl2*^+/+^ mice. ΔEE has been calculated by subtracting the maximum EE in response to NE at 4 °C and at 30 °C. (**D**) Glycaemia of *Bscl2*^+/+^ (WT, black bar) and *Bscl2*^−/−^ (KO, white bar) mice after 5 weeks exposure at 4 °C, 21 °C or 30 °C. (**E**) BAT mass of *Bscl2*^+/+^ (black bar) and *Bscl2*^−/−^ (white bar) mice after five-week exposure at 4 °C, 21 °C or 30 °C. (**F**) Histology (hematoxylin and eosin staining, x600) of *Bscl2*^+/+^ and *Bscl2*^−/−^ BAT. Squares surround typical multi-locular brown adipocytes. Scale bar 100 μm. (**G-J**) Expression of brown adipocyte markers *Ucp1* (**G**), *Dio2* (**H**), *Cidea* (**I**) and *Elovl-3* (**J**) in mice BAT. Bars represent SEM and significant differences between *Bscl2*^−/−^ and *Bscl2*^+/+^ mice in each condition (4 °C, 21 °C, 30 °C) were as follows: *p < 0.05, **p < 0.01, ***p < 0.001. Significant differences between 4 °C or 30 °C acclimated mice and 21 °C acclimated mice of each genotype were as follows: ^*#*^*p* < 0.05, ^*##*^*p* < 0.01, *n* = 6, 6, 9, 9, 6, and 6 per group, respectively.

As a next step, we assessed the ability of Bscl2^−/−^ mice to cope with cold acclimation. Nine-week-old *Bscl2*^+/+^ and *Bscl2*^−/−^ male mice were exposed to cold (one week at 16 °C followed by four weeks at 4 °C), thermoneutrality (five weeks at 30 °C) or control temperature (21 °C). In response to cold exposure, mice of both genotypes maintained their body temperatures at approximately 37 °C during the five weeks of follow-up (Fig. 2A). However, *Bscl2*^−/−^ mice had warmer body temperatures during the first three weeks, consistent with increased basal energy expenditure in cold *Bscl2*^−/−^ mice as compared to wild-type animals (Fig. 2B). *Bscl2*^−/−^ mice remain hyperglycemic at all acclimation temperatures and random fed glycaemia were the highest during cold exposure (Fig. 2D). In parallel, plasma glycerol levels increased in response to cold exposure in both *Bscl2*^−/−^ and *Bscl2*^+/+^ mice, but remained 60% lower in cold-acclimated *Bscl2*^−/−^ mice (Supplemental Fig. 2A,B). In order to assess BAT activation capacity, we measured the energy expenditure (EE) in response to norepinephrine at thermoneutrality and at cold acclimation^20^ (Fig. 2B). Maximum thermogenic capacity, defined by the difference in maximum EE in response to NE between 4 °C and 30 °C, was significantly reduced in *Bscl2*^−/−^ mice as compared to *Bscl2*^+/+^ mice (Fig. 2C).

Interestingly, visual shivering observation revealed that *Bscl2*^+/+^ mice shifted to non-shivering thermogenesis after 10 days at 4 °C, whereas *Bscl2*^−/−^ mice demonstrated a strong and persistent shivering until the end of the experiment (Supplemental Fig. 2C).

We then assessed the remodeling that took place during cold acclimation in BAT. In *Bscl2*^+/+^ mice, BAT mass increased 2-fold upon cold exposure, whereas in *Bscl2*^−/−^ mice there was no significant change (Fig. 2E). The histological analysis of *Bscl2*^−/−^ BAT revealed an abnormal, large LD pattern at 21 °C, which slightly improved at 4 °C with the appearance of a few typical multi-locular brown adipocytes, reflecting some degree of BAT activation (Fig. 2F). The BAT gene expression program in response to cold activation was analyzed by qPCR. Interestingly, *Bscl2* mRNA expression in BAT was not sensitive to temperature changes (Supplemental Fig. 2E). Importantly, the induction of typical thermogenic genes, such as *Ucp1* and *Dio2*, was similar in BAT from *Bscl2*^+/+^ and *Bscl2*^−/−^ mice (Fig. 2G,H). The mRNA levels of the pro-catabolic elongase *Elovl-3* and of the BAT-specific LD-associated protein Cell death-inducing DFFA-like effector a *(Cidea)* were significantly induced by the temperature decrease from 30 °C to 21 °C (a 4-fold and 2.5-fold increase, respectively) in *Bscl2*^−/−^ BAT, although their levels remained lower than those observed in the *Bscl2*^+/+^ mice (Fig. 2I,J). Of note, in our experimental conditions, the control temperature (*i.e.* 21 °C) corresponded to an already activated state of non-shivering thermogenesis. Finally, we assessed the browning of subcutaneous WAT, which also contributes to the thermogenic response to cold. The histological analysis revealed the presence of multi-locular and UCP1 positive beige adipocytes in both *Bscl2*^+/+^ and *Bscl2*^−/−^ WAT (Supplemental 3A,B). *Ucp1*, *Dio2*, *Cidea* and *Elovl-3* mRNA expressions were similarly regulated by temperature changes in *Bscl2*^+/+^ and *Bscl2*^−/−^ inguinal WAT (Supplemental 3C–F). These data suggest that browning of WAT was not impaired in *Bscl2*^−/−^ mice. The beige precursor-specific marker *Tbx15* was similarly expressed in *Bscl2*^+/+^ and *Bscl2*^−/−^ inguinal WAT, suggesting no modification of the precursor pool at cold temperature, whereas WAT-specific genes expression was severely decreased (Supplemental 3G). Altogether, these data indicate that *Bscl2*^−/−^ can cope with cold acclimation but that BAT activation is only partial and less thermogenically competent as compared to *Bscl2*^+/+^ mice.

### β3 agonist CL-316243 failed to activate thermogenesis in *Bscl2* ^−/−^ mice

**Figure 3:**
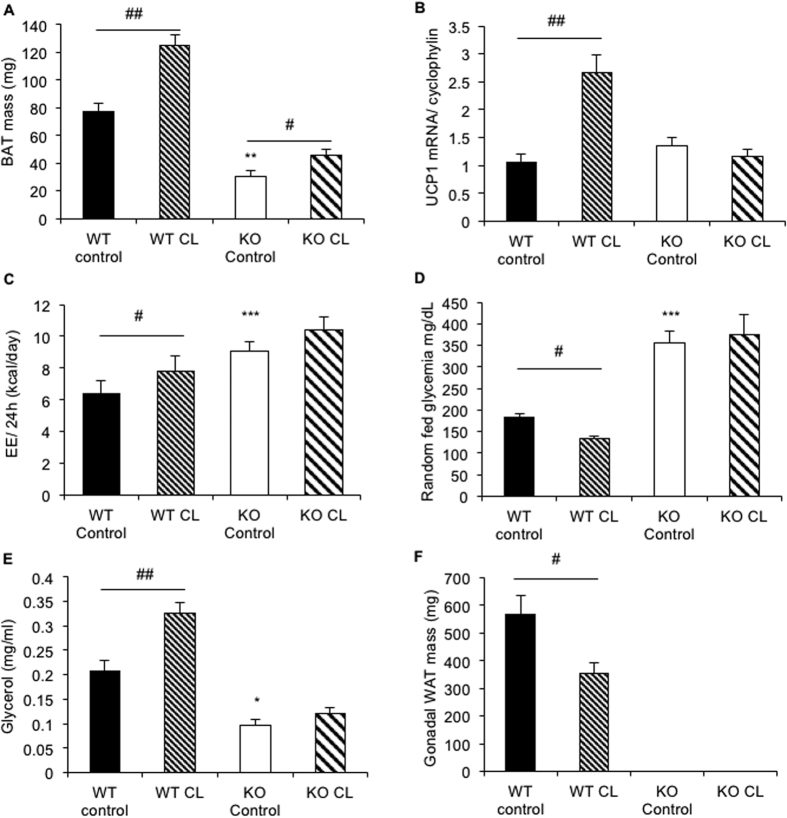
Chronic β3 agonist CL-316243 treatment in *Bscl2*^−/−^ and *Bscl2*^+/+^ mice. *Bscl2*^−/−^ and *Bscl2*^+/+^ mice were treated with CL-316243 during 4 weeks. (**A**) BAT mass in treated (KO CL, stripped white bar) or control (KO control, white bar) *Bscl2*^−/−^ mice, and in treated (WT CL, stripped black bar) or control (WT control, black bar) *Bscl2*^+/+^ mice. (**B**) mRNA level of the brown adipocyte marker *Ucp1* in BAT. (**C**) Energy expenditure was measured using metabolic cages in CL-316243 treated or control *Bscl2*^+/+^ and *Bscl2*^−/−^ mice, after a three-week treatment. (**D**,**E**) Glycaemia and glycerol plasma levels in CL-316243 treated or control *Bscl2*^+/+^ and *Bscl2*^−/−^ mice. (**F**) Mass of gonadal WAT in CL-316243 treated or control *Bscl2*^+/+^ and *Bscl2*^−/−^ mice. Bars represent SEM and significant differences between *Bscl2*^−/−^ and *Bscl2*^+/+^ mice in each condition were as follow: *p < 0.05, **p < 0.01, ***p < 0.001. Significant differences between CL-316243 treated and control condition mice of each genotype were as follow: ^*#*^*p* < 0.05, ^*##*^*p* < 0.01, n = 7; 7; 8; 8 per group.

To ascertain *Bscl2*^−/−^ BAT thermogenesis dysfunction, we then assessed the response of *Bscl2*^−/−^ BAT to a pharmacological stimulus. *Bscl2*^−/−^ and *Bscl2*^+/+^ mice were treated during 4 weeks with a β3 adrenergic agonist (CL-316243). No difference in body weight was noticed in any of the four experimental groups (*data not shown*). BAT mass was increased by the treatment in both genotypes (Fig. 3A). CL-316243 induced significantly the mRNA expression of the thermogenic genes *Ucp1* (Fig. 3B) and the energy expenditure in *Bscl2*^+/+^ mice but not in *Bscl2*^−/−^ mice (Fig. 3C). Consistently with signs of BAT activation, CL-316243 reduced the random fed glycaemia (Fig. 3D) and increased the plasma glycerol levels in *Bscl2*^+/+^ but not in *Bscl2*^−/−^ mice. (Fig. 3E). As a consequence of increased lipolysis, the gonadal fat pad mass was reduced in *Bscl2*^+/+^ mice, while (Fig. 3F) this depot was lacking in *Bscl2*^−/−^ mice^16^. Altogether, these data confirm, that despite signs of remodeling, BAT activation in *Bscl2*^−/−^ mice is defective and fails to induce non-shivering thermogenesis.

### Metabolic inflexibility of *Bscl2* ^−/−^ mice during long-term fasting

**Figure 4:**
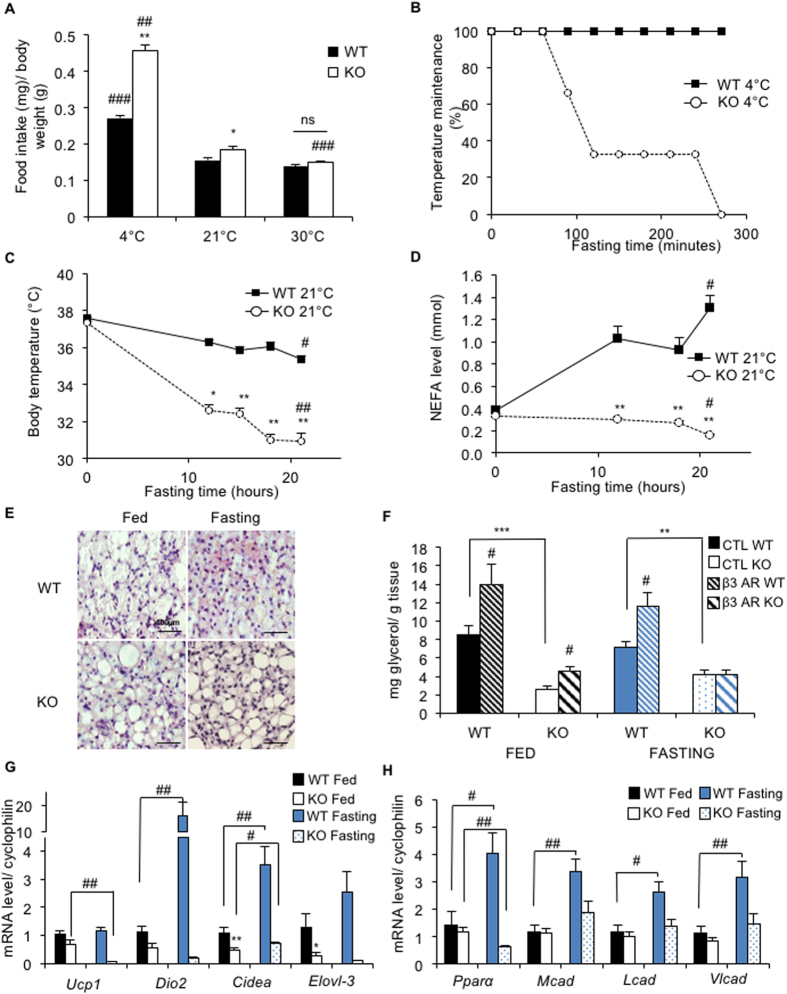
Brown adipose tissue adaptation to long-term fasting. (**A**,**B**) Cold acclimation experiment. (**A**) Food intake of mice housed at 4 °C, 21 °C or 30 °C measured over three consecutive days and normalized to body weight. *B:* Mouse resistance to cold acclimation under fasting condition. The 4 °C acclimated mice were maintained at 4 °C without food. The experiment ended when mouse body temperature loss reached the endpoint of 2.5 °C and the mouse was re-fed. The results are shown as the percentage of mice still in the experiment. (**C**–**H**) Long-term fasting experiments: *Bscl2*^+/+^ (WT, black bar, solid line) and *Bscl2*^−/−^ mice (KO, white bar, dashed line) were maintained without food at control temperature (21 °C) until the body temperature endpoint was reached (body temperature lower than 31 °C). Body temperature (**C**), and NEFA level (**D**) monitored every 3 h or 6 h. (**E**) Histology (hematoxylin and eosin staining x600) of *Bscl2*^+/+^ (black bar) and *Bscl2*^−/−^ (white bar) BAT. Scale bar 100 μm. (**F**) *Ex vivo* measurement of glycerol release by BAT explants from mice in the random fed or 6h-fasting state (in blue). Measurements were performed after 2 h incubation at 37 °C with (dashed bar) or without (filled bar) induction of lipolysis by the ß3AR agonist CL-316243. (**G**) Expression of *Ucp1*, *Dio2*, *Pgc1a*, *Cidea* and *Elovl-3* mRNA in the fed (black) or fasting (blue) state. (H) Expression of fatty acid oxidation genes in BAT. n = 6 per group. Bars represent SEM. Significant differences between *Bscl2*^−/−^ and *Bscl2*^+/+^ mice in each condition (4, 21, 30 °C in figure A; and 0, 12, 15, 18, 21 h fasting in figures (**C**–**H**) were as follows: *p < 0.05, **p < 0.01, ***p < 0.001. Significant differences between the different states (between 4 °C or 30 °C acclimated mice and 21 °C acclimated mice in figure A; between a fed state (0 h) and a 21 h-fasting in figures (**C**,**D**,**G**,**H**); between a control state and a β3-agonist stimulated state in figure (**F**) of each genotype (WT, KO) were as follows: ^#^p < 0.05, ^##^p < 0.01, ^###^p < 0.001.

During the cold acclimation experiment (detailed in Fig. 1), we observed a 100% increase in food intake (Fig. 4A) in *Bscl2*^−/−^ mice compared with *Bscl2*^+/+^ mice. In order to determine the importance of this phenomenon in the cold adaptation, we repeated the experiment in fasted *Bscl2*^−/−^ mice. Interestingly, 4-week cold-acclimated *Bscl2*^−/−^ mice displayed a rapid drop in their body temperature upon fasting (Fig. 4B). After a 4.5-h-fast, all *Bscl2*^−/−^ mice reached the temperature endpoint (*i.e.* 2.5 °C body temperature loss), whereas all *Bscl2*^+/+^ mice maintained their body temperature. In parallel, glycaemia quickly dropped from 500 mg/dL to 180 mg/dL during fasting in *Bscl2*^−/−^ mice, reaching the same level as observed in *Bscl2*^+/+^ mice (Supplemental Fig. 4A). Together, these results suggest that *Bscl2*^−/−^ mice dealt with cold exposure through high-energy uptake.

As a next step, we characterized the consequences of long-term fasting on BAT function at control temperature (21 °C) in *Bscl2*^−/−^ mice. First of all, we noticed that *Bscl2*^−/−^ mice are intolerant to fasting, as the experiment had to be stopped after a 21h-fast because the body temperature endpoint was reached (lower than 31 °C). At the end of the experiment, *Bscl2*^−/−^ mice demonstrated abnormal behavior (prostrated in the back of the cage), and low activity, suggesting a state of torpor^21^. Body temperatures of *Bscl2*^−/−^ mice significantly decreased from 12 h of fasting onward (Fig. 4C). Strikingly, *Bscl2*^−/−^ mice did not display the expected increase in NEFA and ketone body plasma levels upon fasting (Fig. 4D, Supplemental Fig. 4B).

As expected^22^, BAT mass decreased during fasting in both genotypes (Supplemental Fig. 4C). Hematoxylin and eosin staining of BAT sections showed a reduced lipid content in *Bscl2*^+/+^ BAT, whereas no histological modifications were found in the *Bscl2*^−/−^ BAT (Fig. 4E). The functional consequence of fasting on *Bscl2*^−/−^ BAT activation was assessed by comparing *ex vivo* lipolysis from BAT explants of fed or 6-h fasted mice (Fig. 4F). Interestingly, the induction of lipolysis by CL-316243, about 75% in the fed state, was abolished under fasting conditions in the *Bscl2*^−/−^ explants (Fig. 4F). In order to better characterize this discrepancy, we studied BAT gene expression profile in the fed and fasting states. Although *Ucp1* expression remained stable, *Dio2* and *Cidea* mRNA levels were strongly increased (16-fold and 3.5-fold, respectively) in response to fasting in *Bscl2*^+/+^ BAT (Fig. 4G). In contrast, the mRNA levels of these thermogenic genes (*Ucp1*, *Dio2*, *Cidea* and *Elolv-3*) were strongly decreased in *Bscl2*^−/−^ *vs Bscl2*^+/+^ BAT in the fasting state. In addition, the expression of fatty acid oxidation genes encoding the peroxysome proliferator-activated receptor itself *(Pparα)*, and its targets, the medium-chain acyl-CoA dehydrogenase *(Mcad)*, the long-chain acyl-CoA dehydrogenase *(Lcad)* and the very long-chain acyl-CoA dehydrogenase *(Vlcad)*, which were similarly expressed in *Bscl2*^+/+^ and *Bscl2*^−/−^ BAT in the fed state, failed to increase in response to fasting in *Bscl2*^−/−^ BAT (Fig. 4H). Thus, the defect of *Bscl2*^−/−^ BAT activity is strongly reinforced upon fasting with a decreased response to adrenergic stimulus. This default is associated with defective metabolic flexibility in *Bscl2*^−/−^ mice.

### *Bscl2* ^−/−^ BAT is insulin resistant and has reduced glucose uptake

**Figure 5:**
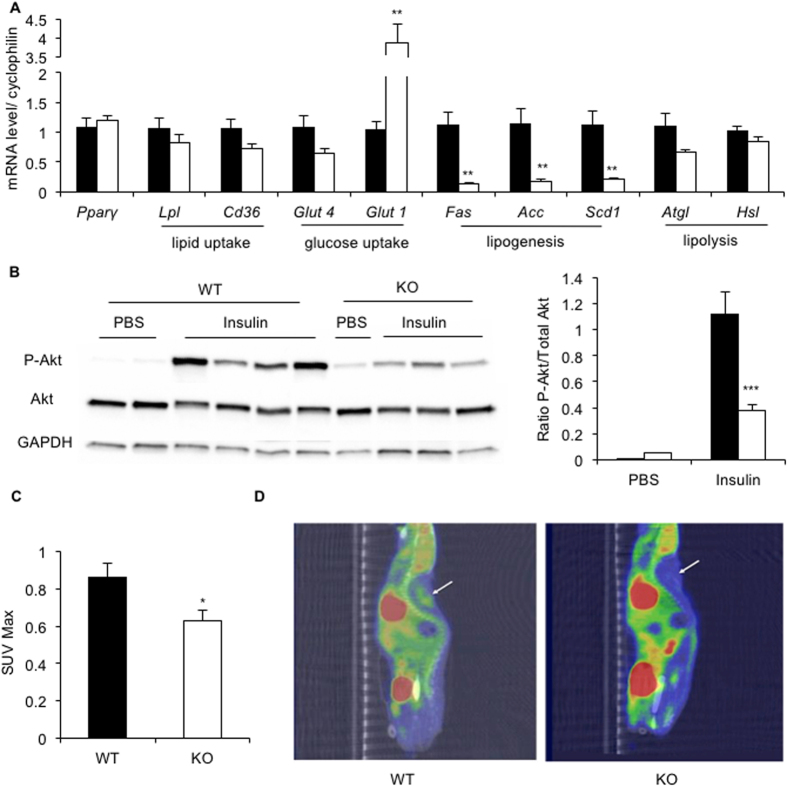
Insulin resistance in BAT from *Bscl2*^−/−^ mice. (**A**) Gene expression profile in BAT of *Bscl2*^+/+^ and *Bscl2*^−/−^ mice housed at control temperature (21 °C). Gene expression was normalized to cyclophilin expression. (**B**) Insulin signaling: Western Blot quantification of phosphorylated Akt and total Akt in BAT of *Bscl2*^+/+^ and *Bscl2*^−/−^ mice in response to insulin or PBS injection (n = 8 per group for insulin treated and n = 3 for saline control). (**C**–**D**) Quantification of ^18^F-FDG uptake in BAT of *Bscl2*^+/+^ and *Bscl2*^−/−^ mice in the basal condition during PET acquisition, n = 7 per group (**C**). CT-PET coupled imaging of ^18^F-FDG uptake in *Bscl2*^−/−^ (right picture) and *Bscl2*^+/+^ mice (left picture) (**D**). A region of interest (ROI), indicated by the arrow, was manually determined to encompass BAT at the interscapular region of the neck, using the co-registered PET and CT images. Bars represent SEM, and significant differences between *Bscl2*^−/−^ and *Bscl2*^+/+^ mice in each condition (4 °C, 21 °C, 30 °C) were as follows: *p < 0.05, **p < 0.01, ***p < 0.001.

Finally, in order to understand the molecular mechanisms involved in the lowest ability of seipin-deficient BAT to ensure non-shivering thermogenesis, we performed an extended BAT gene expression profile (Fig. 5A). Seipin deficiency did not affect the expression level of the adipogenesis master regulator *Pparγ*, and of genes involved in lipid uptake (lipoprotein lipase *(Lpl)* and cluster of differentiation (*Cd36)*) or lipolysis (adipose triglyceride lipase *(Atgl)* and hormone-sensitive lipase *(Hsl*)) (Fig. 5A). The mRNA level of the insulin-sensitive glucose transporter *Glut4* was unaffected in *Bscl2*^−/−^ BAT, whereas *Glut1* expression increased 4-fold. Importantly, the expression levels of the lipogenic genes encoding the fatty acid synthase *(Fas)*, acetyl-CoA carboxylase *(Acc)* and stearoyl-CoA desaturase-1 (*Scd1)* were markedly decreased (Fig. 5A). Because these genes are induced by insulin, we tested insulin signaling *in vivo* in BAT. In accordance with BAT insulin resistance, insulin-induced Akt phosphorylation was strongly decreased in *Bscl2*^−/−^ BAT (Fig. 5B). At the same temperature acclimation, we performed PET scan imaging in unstimulated condition and observed a significant decrease in ^18^F-Fluorodeoxyglucose uptake in *Bscl2*^−/−^ BAT (Fig. 5C,D). Taken together, these results show that *Bscl2*^−/−^ BAT displays a reduced glucose uptake capacity and an insulin resistant profile.

## Discussion

Seipin deficiency is responsible for the most severe form of BSCL. *Bscl2*^−/−^ mice display an extended loss of WAT and a milder atrophy of BAT and thus represent a valuable model for deciphering the pathophysiology of BSCL. Although the consequences of seipin deficiency in WAT have been studied^14^,^15^,^16^. The functionality of BAT in this lipodystrophic model has not been thoroughly assessed. Using a brown adipocyte cell-line, we show that seipin knockdown did not affect insulin sensitivity and β3 adrenergic stimulated lipolysis or oxygen consumption. On the other hand, upon cold or β3 adrenergic stimulation *in vivo*, *Bscl2*^−/−^ BAT failed to induce properly a thermogenic response. We highlight that these defaults might be due, at least partially, to the lack of energetic substrates from WAT, and to a marked insulin resistance in BAT.

Because *Bscl2*^−/−^ mice display BAT atrophy^14^,^15^,^16^ and because seipin is essential for white adipocyte differentiation, we addressed the question of a cell-autonomous role for seipin in brown adipocytes. We demonstrate here, using seipin knock-down in a brown pre-adipocyte cell line, that seipin deficiency did not alter the induction of the adipogenic genes such as *Pparγ, aP2*, lipogenic or lipolytic genes. On the other hand, seipin knockdown prevented full activation of the thermogenic program (*Ucp1, Dio2, Elovl3*) whereas seipin-deficient cells had normal basal oxygen consumption and respond fully to insulin and adrenergic stimulations. We obtained similar results using primary brown pre-adipocytes differentiated *in vitro*. During the reviewing of this article, Zhou *et al*. reported that seipin is not required for brown adipogenesis using immortalized neonatal brown pre-adipocytes^23^. However, in their model, seipin-deficient cells displayed a higher expression of *Ucp1* and increased oxygen consumption. The different models used might explain this discrepancy: we have used either knockdown in immortalized preadipocyte cell lines, or primary pre-adipocytes isolated from BAT of 4- week old knockout mice while they had used immortalized brown preadipocytes isolated from knockout neonatal mice. Altogether, it can be concluded that seipin *per se* is not essential for brown adipocyte differentiation and functionality *in vitro*.

Furthermore, although cold-acclimated *Bscl2*^−/−^ mice display altered expression of white adipocyte markers, the induction of the thermogenic gene program, *i.e.* browning, was not affected. This indicates an effective recruitment of beige adipocytes, highlighting the discrepancy between the consequences of seipin deficiency in white adipocyte *vs* thermogenic brown/beige adipocyte differentiation. Recent studies have shown that brown, beige and white adipocytes are derived from different lineages, suggesting that seipin may have distinct roles depending on the adipocyte origin^24^. However, during the differentiation of seipin knockdown 3T3-L1 pre-adipocytes, there was a transient overexpression of *Ucp1* mRNA^19^, raising the hypothesis that seipin-deficient adipocytes could acquire features of beige adipocytes^25^.

BAT from *Bscl2*^−/−^ mice has been previously shown to display abnormal LD morphology^15^. However, functionality of this BAT has not been explored. During the preparation of this manuscript, Ebihara *et al*. reported that in *Bscl2*^−/−^ rats, 24-h-cold exposure induced a similar thermogenic program as observed in *Bscl2*^+/+^ rats^26^. In the present study, using long-term cold acclimation, we show that the BAT from *Bscl2*^−/−^ mice can indeed switch on the thermogenic gene program under physiological stimuli such as cold exposure. However, this was not sufficient to allow the non-shivering transition under cold activation in *Bscl2*^−/−^ mice. Furthermore, the maximal thermogenic capacity assessed by the gold standard method, *i.e* oxygen consumption after norepinephrine injection, is reduced in *Bscl2*^−/−^ mice at 4 °C. Finally, β3 adrenergic stimulation by chronic treatment with CL-316243 fails to increase oxygen consumption and *Ucp1* mRNA induction in *Bscl2*^−/−^ mice, despite an increase of BAT mass. Altogether, these data indicate that cold or β3 adrenergic stimulations could induce only a partial BAT remodeling in *Bscl2*^−/−^ mice, that fails to properly activate non-shivering thermogenesis.

Whereas *Bscl2*^−/−^ mice cope with cold acclimation when energy is supplied, their body temperature dropped in less than five hours when fasting. These dramatic results led us to better characterize the energetic flux and supply in these mice. At control temperature (21 °C), *Bscl2*^−/−^ mice were intolerant to fasting and entered into torpor after 21 h of fasting. Upon fasting, plasma glucose levels dropped rapidly (*data non shown*), plasma NEFA levels did not raise and ketone body production was ineffective, which was consistent with the fact that the *Pparα* pathway failed to be activated in the liver (*data not shown*). Interestingly, BAT impairment worsened under fasting conditions. Thermogenic and *Pparα* target gene expression levels were reduced, and β3-adrenergic stimulated lipolysis is blunted. In this context of generalized lipodystrophy, fasting state that quickly leads to a lack of energetic substrate availability strongly alters BAT function. In accordance, during cold exposure, *Bscl2*^−/−^ mice compensate for the decreased NEFA availability, due to the defective white adipose tissue, by a higher energy intake. Beyond abnormal substrate management, WAT loss in the lipodystrophic model also results in decreased leptin and adiponectin plasma levels^16^. Leptin was shown to enhance thermogenesis *via* the adrenergic pathway and to play a major role in regulating fasting-induced torpor^27^,^28^, while adiponectin is known to be correlated to the β3-adrenergic response in adipose tissue and to participate to fasting and refeeding adaptation^29^. During fasting and cold exposure, plasma leptin levels in *Bscl2*^−/−^ mice decreased to an undetectable level (*data not shown*)^29^. Thus, we can hypothesize that in our model, the lack of WAT, as an energetic substrate supplier and an adipokines producer, might play a key role in the BAT dysfunction reported here. Indeed, Zhou *et al*.^23^ demonstrated that specific deletion of seipin in myf-5^+^ precursor cells led to BAT atrophy but did not alter the BAT thermogenic response to cold acclimation, supporting the hypothesis that seipin deficiency in BAT can not explain *per se* the altered thermogenic response that we are reporting here. Altogether, these data support that constant energy supply and adequate adipokines levels are necessary for physiological BAT activation.

One of the strongest defects detected in *Bscl2*^−/−^ BAT is the insulin resistant state, revealed by the reduced insulin signaling and the lower expression of insulin target genes, in particular, the lipogenic genes. This also probably explains the decreased glucose uptake in *Bscl2*^−/−^ BAT. This defect is probably due to a default in insulin-regulated Glut4 glucose transporter activity, since the expression of the non insulin-dependent glucose transporter Glut1 is increased in Bscl2^−/−^ BAT. This insulin-resistant pattern recapitulates features of mice lacking insulin receptors in BAT (BATIRKO mice), in which knowkdown of insulin signaling in BAT decreased lipogenesis without altering *Ucp1* expression^30^. Although cold adaptation was not investigated in BATIRKO mice, previous studies have shown that insulin resistance in BAT is associated with decreased glucose uptake in response to acute cold exposure^31^. The increased macrophage infiltration and the ceramide accumulation reported in *Bscl2*^−/−^ BAT could largely contribute to this insulin resistance^17^. It is important to bear in mind that SKD brown adipocytes are fully insulin responsive, therefore, we would hypothesize that the insulin resistance is not due to a cell autonomous role of seipin in BAT but must rather be a consequence of WAT failure.

From a clinical perspective, as BAT activation has largely been shown to improve insulin resistance associated with obesity^32^, one could reasonably wonder whether BAT represents a suitable therapeutic target to cure the metabolic complications associated with lipodystrophy. Despite the lack of a marked cell autonomous effect of seipin in the brown adipocyte, our results show that cold acclimation does not improve the *Bscl2*^−/−^ mouse metabolic profile. BAT insulin resistance and decreased BAT glucose uptake may certainly play a central role in the lack of any beneficial metabolic effect. Regarding our understanding of seipin biology, several works intended to understand whether the metabolic effect of seipin deficiency are white adipocyte centered or cell autonomous. Whereas hepatic seipin deficiency does not affect TG liver storage in mice^33^, seipin mutation in the salivary gland in drosophila alters the lipid storage in this organ in a cell autonomous manner^34^. Here, we would hypothesize that the BAT dysfunction *in vivo* is primarily due to the failure of WAT, as an energetic substrate provider and adipokine-producing organ. Therefore, the WAT/BAT dialogue is a key component of BAT integrity in guaranteeing its response to insulin and cold-activated adrenergic signals.

## Methods

### Animals, temperature acclimation, fasting and β3-agonist experiments

*Bscl2*^−/−^ mice were generated as previously described^16^ and were housed at 21 °C with a 12:12 h light-dark cycle with free access to food and water. All the experimental procedures were approved by the regional ethic committee (CEEA – Pays de la Loire, France) according to the Directive 2010/63/EU of the European Union. All animal experimentations were carried out in accordance with the relevant guidelines and recommendations. For the acclimation experiments, nine-week-old littermate *Bscl2*^+/+^ and *Bscl2*^−/−^ mice were singly housed in a thermoregulated chamber (Bio-Concept Technologies, Changé, France) and maintained in a cold environment (one week at 16 °C followed by four weeks at 4 °C), a thermoneutral environment (five weeks at 30 °C) or at a control temperature (21 °C). Shivering was estimated by daily observation at 9:00 A.M. and at 7:00 P.M. using scores of 8 (strong shivering, mice do not leave nest), 6 (strong shivering but movement in the cage), 4 (shivering and movement) or 2 (mild shivering and movement). Body temperature was measured using a rectal probe (Bioseb, Vitrolles, France). Food intake was measured on week 4 over three days. After five weeks of acclimation, mice were killed in a random fed state and organs were collected. Assessment of norepinephrine-induced energy expenditure was performed in anesthetized mice (pentobarbital, 70 mg/kg), after 4 weeks acclimation at 30 °C or 4 °C. Energy expenditure was measured using the physiocage system (Panlab, Barcelona, Spain), in a 33 °C chamber. At t = 25 min, mice were injected with norepinephrine (1 mg/kg). For the long-term fasting experiment at control (21 °C) temperature, food was removed at 20:00 and *Bscl2*^+/+^ and *Bscl2*^−/−^ mice were killed after 21 hours fasting. For the β3-agonist experiment, CL316243 (1mg/kg/day) was delivered by osmotic pumps (Model 1004; Alzet Inc) during 4 weeks to *Bscl2*^+/+^ and *Bscl2*^−/−^ mice. Energy expenditure was measured using the physiocage system (Panlab, Barcelona, Spain) as previously described^16^. ΔEE has been calculated by subtracting the values of EE at 54-60-66 minutes at 30 °C to those obtained at 4 °C.

### Blood biochemistry

Glucose and ketone bodies (β-hydroxybutyrate) were measured using a glucometer (Freestyle Optium Neo, Abbot, Rungis, France) with glucose or ketone body test strips, respectively. Plasma non-esterified fatty acid (NEFA) and glycerol concentrations were measured with a NEFA-HR (2) kit (Wako Diagnostics, Richmond, CA, USA) and a Glycerol kit (Biomérieux, Marcy l’Etoile, France).

### *In vivo* and *ex vivo* insulin signaling

For *in vivo* insulin signaling, twelve-week-old *Bscl2*^+/+^ and *Bscl2*^−/−^ mice were fasted for 4 h and a single dose of 1 UI/kg of human recombinant insulin (Umuline rapide, Eli Lilly, Suresnes, France) was administered by intravenous injection. Mice were killed 5 min after injection and brown fat pads were removed. For *in vitro* insulin signaling, cells were starved during 18 h and 100 nM insulin was added. Cells were harvested after 5 min and proteins were extracted. Western blots were performed using P-Akt and Pan-Akt antibodies (Cell Signaling Technology, Danvers, MA, USA).

### Micro-PET/CT (positron emission tomography computed tomography)

Two-hour-fasted animals were anesthetized with isoflurane (1.5–3%) in an oxygen/nitrous oxide mix (0.6 L/min) via spontaneous breathing, and were placed on a warming pad (37 °C). No exogenous insulin was injected during PET exams. A microcatheter was surgically inserted in the tail vein prior to animal installation in a dedicated preclinical micro-PET/CT scanner (Inveon®, Siemens Medical Solutions, Knoxville, TN, USA). When blood glucose ranged between 5.5 and 9.9 mmol/L, a 60 min PET acquisition was started along with a 10 s tail vein injection of ^18^F-FDG (15–20 MBq in 50–100 μl). For BAT assessment, data were summed on a static frame and analyzed using the open source software OsiriX (http://www.osirix-viewer.com/) with the Standardized Uptake Value (SUV) plugin. A region of interest (ROI) was manually determined to encompass BAT at the interscapular region of the neck using the co-registered PET and CT images. Regarding the injected dose, body-weight and decay correction, the maximum SUV (SUV Max) was determined within the ROI.

### Histology

Liver, BAT and inguinal WAT sections were fixed with 10% formalin. Paraffin-embedded 7 μm sections were then stained with hematoxylin and eosin according to standard laboratory protocols. In WAT sections, Ucp1 staining was performed using UCP1 antibody (ab10983, Abcam) diluted to 1/500.

### Brown preadipocytes

For knockdown experiments, brown preadipocytes were isolated from neonatal BAT from one-day-old *Bscl2*^+/+^ mice. Primary brown preadipocytes were then infected with freshly collected SV40 T retrovirus-containing supernatants with 8 μg/ml polybrene and cells were selected by G418 (450 μg/ml). Immortalized *Bscl2*^+/+^ brown preadipocytes were infected using lentivirus (Smartvector lentiviral shRNA, GE Healthcare, Velizy-Villacoublay, France) containing either a non-targeted shRNA sequence (NTC cell-line) or an shRNA against seipin (SKD cell-line). For differentiation assays, cells were plated at 70,000 cells/ml with 1 nM T3 and 20 nM insulin. After 24 h, IBMX (500 μM) and dexamethasone (1 μM) were added to the media for 24 h before changing back to the previous media containing T3 and insulin only. Oil Red O and Bodipy staining were performed as previously described^16^. To isolate primary adipocytes, BAT from 4 weeks-old *Bscl2*^+/+^ or *Bscl2*^−/−^ mice was removed and digested with collagenase II (Sigma Aldrich, Saint-Quentin Fallavier, France). The stromal vascular fraction containing preadipocytes was cultured in DMEM with 20% fetal serum bovine (FBS). For differentiation assays, cells were cultured with 1 nM T3, 60 nM insulin, and 100 μg/ml sodium ascorbate (Sigma Aldrich, Saint-Quentin Fallavier, France) during 7 days.

### *In vitro* and *ex vivo* lipolysis

Cells were incubated in the presence or absence of a β3-specific agonist, CL-316243, 0.1 μM (Sigma Aldrich, Saint-Quentin Fallavier, France). After 2 h at 37 °C, the medium was collected and glycerol levels were determined using the Free Glycerol Reagent (Sigma-Aldrich, Saint-Quentin Fallavier, France). Inguinal WAT was removed and cut into 10 mg fat pads. Fat pads were incubated in low glucose DMEM with 2% BSA in the presence or absence of 1 μM CL-316243 for 2 h at 37 °C. An explant from each animal was allocated to each *ex vivo* treatment group. The medium was then collected for glycerol level determination.

### Seahorse experiment

Cells were plated and differentiated during 7 days in SeaHorse XF24 cell culture microplates (Agilent Technology, Interchim, Montluçon, France) and the run was performed with the SeaHorse XF Analyzer. Oxygen consumption was measured in the basal condition and after adding 1 μM CL-316243.

### Western blot, and RNA analysis

For western blot analyses, we utilized phospho-Akt and pan-Akt antibodies (Cell Signaling) and UCP11-A antibody from Alpha Diagnostic International (Interchim, Montluçon, France). RNA expression was analysed as previously described^16^.

### Statistical analysis

All data were reported as means  ±  SEM (standard error of means). Data sets were analyzed for significance using the non-parametric Mann–Whitney U test or Wilcoxon test, and when mentioned in the figure legend, Kruskal-Wallis or two-way ANOVA analyses; Energy expenditure (EE) data were adjusted by mean bodyweight using analysis of covariance (ANCOVA) with weight as the covariate. *p < 0.05, **p < 0.01 and ***p < 0.001; ^#^*p* < 0.05, ^##^*p* < 0.01 and ^###^*p* < 0.001.

## Additional Information

**How to cite this article**: Dollet, L. *et al*. Seipin-deficiency alters brown adipose tissue thermogenesis and insulin sensitivity in a non-cell autonomous mode. *Sci. Rep.* **6**, 35487; doi: 10.1038/srep35487 (2016).

## Supplementary Material

Supplementary Information
