# Supplementary material for: Seipin deficiency alters brown adipose tissue thermogenesis and insulin sensitivity in a non-cell autonomous mode
Source: Sci Rep. 2016 Oct 17;6:35487. doi: 10.1038/srep35487 (PMC5066230; doi:10.1038/srep35487)

Seipin-deficiency alters brown adipose tissue thermogenesis and insulin sensitivity in a non-cell autonomous mode

L. Dollet, J. Magré, M Joubert, C. Le May, A. Ayer, L. Arnaud, C. Pecqueur, V. Blouin, B. Cariou, X. Prieur

Supplemental materials

Supplemental Fig. 1. Effects of seipin deficiency on brown adipocyte differentiation. Differentiation assays in *Bscl2*$^{+/+}$ and *Bscl2*$^{-/-}$ murine primary brown preadipocytes. *A:* Bodipy staining in 7 days-differentiated *Bscl2*$^{+/+}$ (WT) and *Bscl2*$^{-/-}$ (KO) primary brown adipocytes. Scale bar 1000µm.

B: Gene expression profile in non-differentiated (WT and KO CTL) and differentiated (WT and KO DMI) primary brown adipocytes. n=6 primary adipocyte preparations for each experimental condition. *C:* Western-blot quantification of phosphorylated-AKT and total AKT in differentiated *Bscl2*$^{+/+}$ (WT) and *Bscl2*$^{-/-}$ (KO) primary brown adipocytes, in absence or after insulin treatment. n=4 primary adipocyte preparations for each experimental condition.

Significant differences between *Bscl2*$^{-/-}$ and *Bscl2*$^{+/+}$ primary adipocytes in each condition (control, DMI) were as follows: *$p<0.05$, **$p<0.01$, ***$p<0.001$. Significant differences between the different conditions (control, DMI, insulin) in each genotype were as follows: #$p<0.05$; ##$p<0.01$; ###$p<0.001$).

Supplemental Fig. 2. Adaptation of *Bscl2*$^{+/+}$ and *Bscl2*$^{-/-}$ mice to temperature challenges and pharmacological treatment. *A:* Glycerol levels of *Bscl2*$^{+/+}$ (WT, black bar) and *Bscl2*$^{-/-}$ (KO, white bar) mice after 5 weeks exposure at 4°C, 21°C or 30°C. *B:* Variation of glycerol levels between 21°C and 4°C acclimated mice (in %). *C:* Visual quantification of shivering intensity during the first 21 days at 4°C. *D:* Final body weight of *Bscl2*$^{+/+}$ and *Bscl2*$^{-/-}$ mice. *E:* Seipin expression is not modulated by housing temperature (4°C, 21°C or 30°C) in *Bscl2*$^{+/+}$ BAT.

Significant differences between *Bscl2*$^{-/-}$ and *Bscl2*$^{+/+}$ mice in each condition (4°C, 21°C, 30°C) were as follows: *$p<0.05$, **$p<0.01$, ***$p<0.001$. Significant differences between 4°C or 30°C acclimated mice and 21°C acclimated mice of each genotype were as follows: #$p<0.05$; ##$p<0.01$; ###$p<0.001$).

Supplemental Fig. 3. Response of *Bscl2*$^{-/-}$ inguinal WAT to a cold stimulus. *A:* Hematoxylin and eosin staining (x600) and *B:* UCP1 staining (x600) in *Bscl2*$^{+/+}$ and *Bscl2*$^{-/-}$ inguinal WAT after five-

week exposure at 4°C, 21°C or 30°C. Scale bar 100µm. *C-F:* Gene expression of *Ucp1* (C), *Dio2* (D), *Cidea* (E) and *Elovl-3* (F) in inguinal WAT. *G*: Expression of the beige precursor marker *Tbx15* mRNA and typical white adipocyte genes in inguinal WAT after five-week exposure at 4°C. Gene expression was normalized to cyclophilin expression. Bars represent SEM. Significant differences between *Bscl2*$^{-/-}$ and *Bscl2*$^{+/+}$ mice in each condition (4°C, 21°C, 30°C) were as follows: **p*<0.05, ***p*<0.01, ****p*<0.001. Significant differences between 4°C or 30°C acclimated mice and 21°C acclimated mice of each genotype were as follows: #*p*<0.05; ##*p*<0.01; ###*p*<0.001).

Supplemental Fig. 4. Effect of fasting on *Bscl2*$^{-/-}$ mice metabolism. *A:* Mice resistance to cold acclimation under fasting conditions. 4°C acclimated *Bscl2*$^{+/+}$ (WT, black bar) and *Bscl2*$^{-/-}$ (KO, white bar) mice were maintained at 4°C without food, and glycemia was measured at the beginning and at the end of the experiment. *B:* Ketones bodies level of *Bscl2*$^{+/+}$ (solid line) and *Bscl2*$^{-/-}$ (dashed line) mice during the 21h-fasting experiments. *C:* Mass of *Bscl2*$^{+/+}$ (black bar) and *Bscl2*$^{-/-}$ (white bar) BAT in random fed state or after 21h of fasting. Bars represent SEM. Significant differences between *Bscl2*$^{-/-}$ and *Bscl2*$^{+/+}$ mice in each condition (0, 12, 15, 18, 21 h fasting) were as follows: *p<0.05, **p<0.01, ***p<0.001. Significant differences between fed state and 21 h fasting state of each genotype (WT, KO) were as follows: #p<0.05, ##p<0.01, ###p<0.001.

Supplemental Figure 1

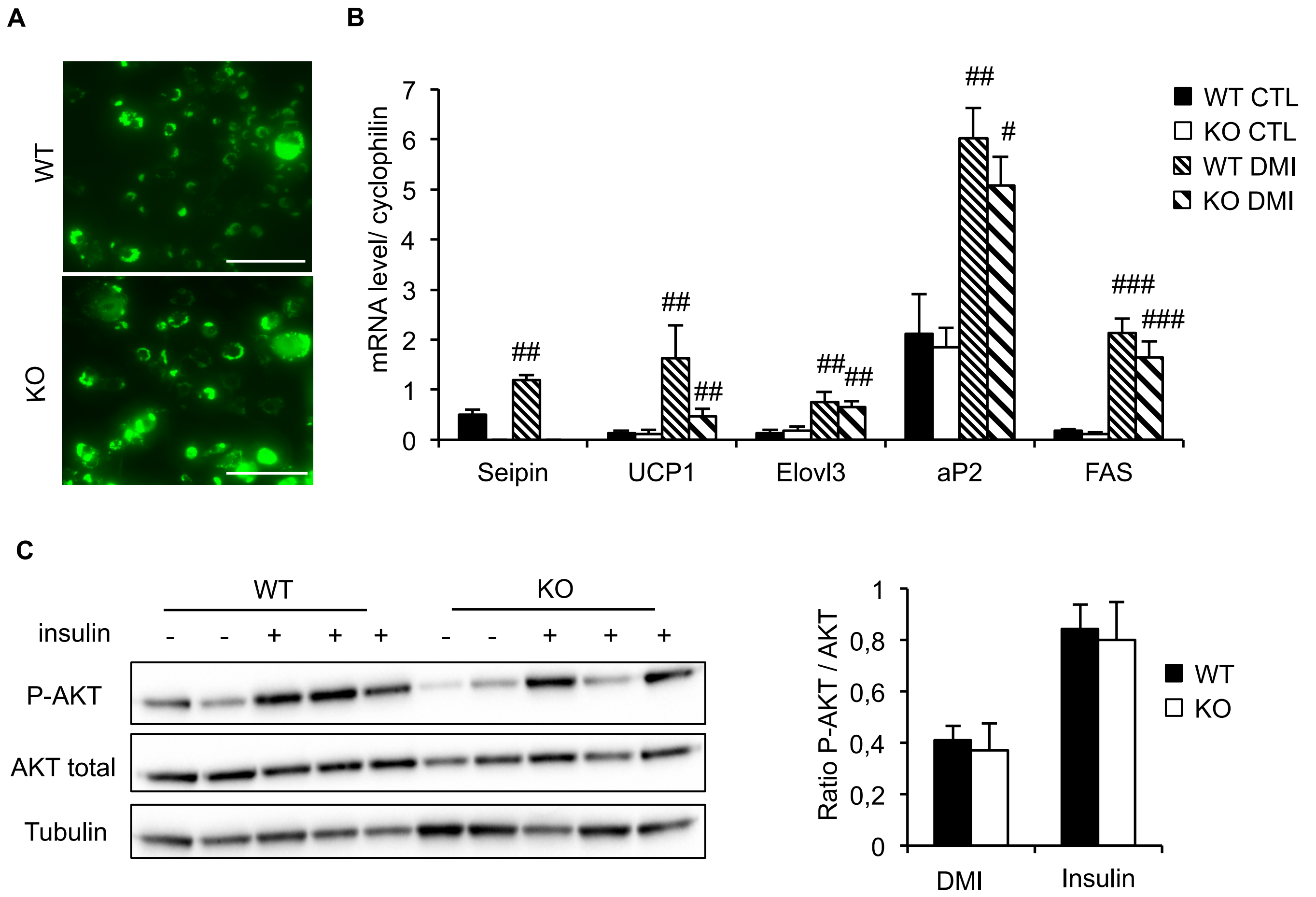

Supplemental Figure 2

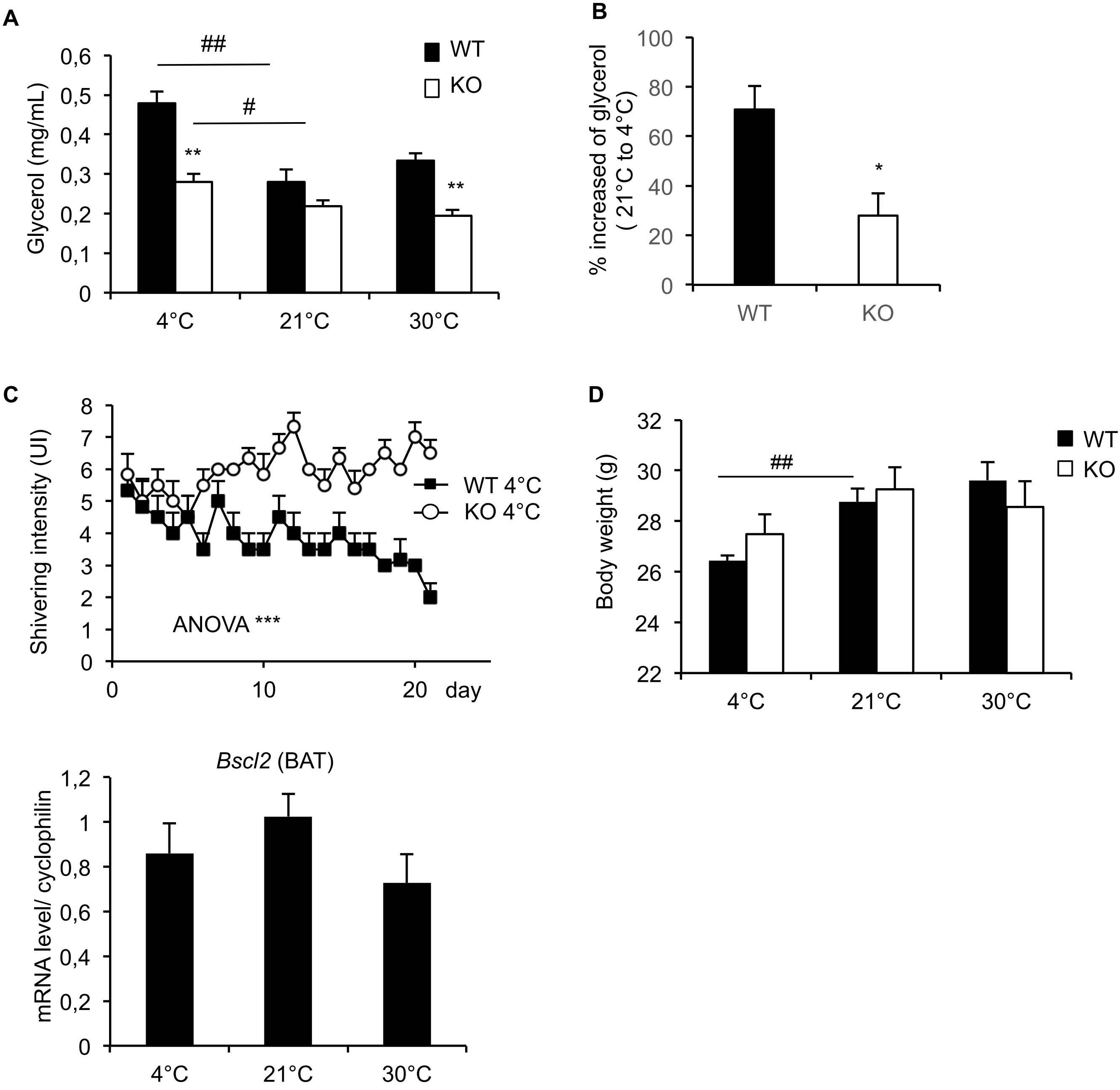

Supplemental figure 3

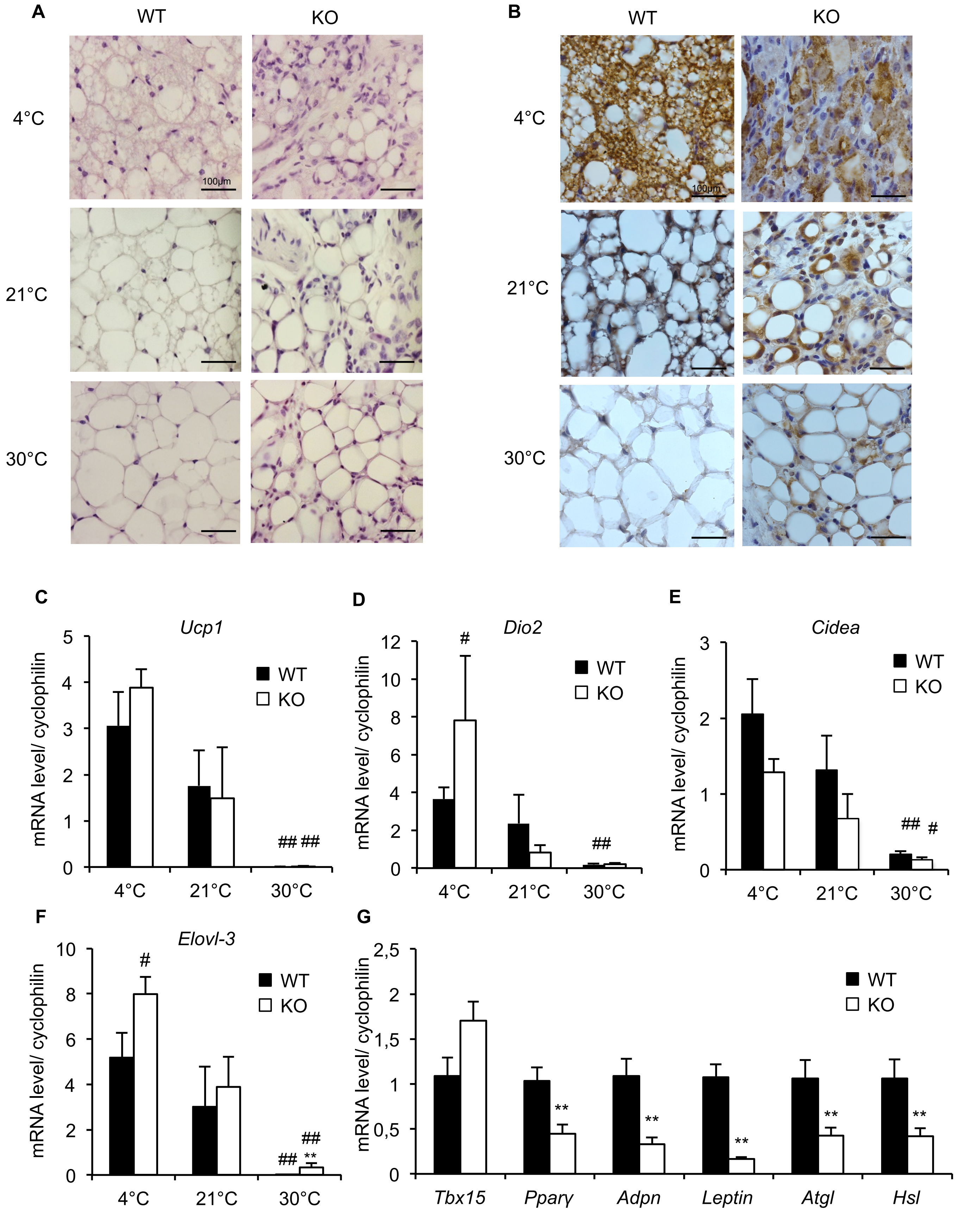

Supplemental Figure 4

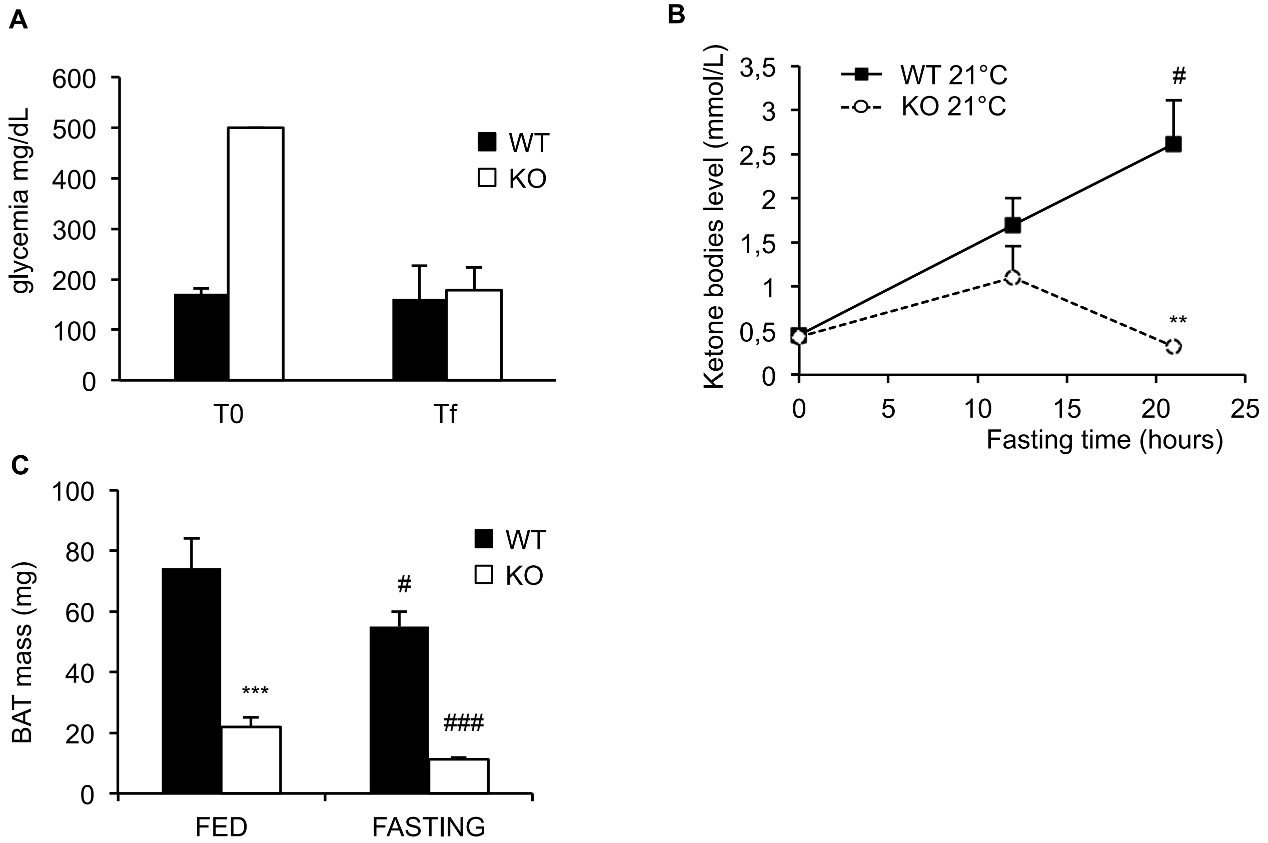

Supplement: Supplementary Information [file srep35487-s1.pdf]
